# Duchenne Muscular Dystrophy Newborn Screening, a Case Study for Examining Ethical and Legal Issues for Pilots for Emerging Disorders: Considerations and Recommendations

**DOI:** 10.3390/ijns4010006

**Published:** 2018-01-25

**Authors:** Michele A. Lloyd-Puryear, Thomas O. Crawford, Amy Brower, Kristin Stephenson, Tracy Trotter, Edward Goldman, Aaron Goldenberg, R. Rodney Howell, Annie Kennedy, Michael Watson

**Affiliations:** 1 Parent Project Muscular Dystrophy, 401 Hackensack Avenue, 9th Floor, Hackensack, NJ 07601, USA; 2 Departments of Neurology and Pediatrics, Johns Hopkins University, Baltimore, MD 21218, USA; 3 American College of Medical Genetics and Genomics, Bethesda, MD 20814, USA; 4 Muscular Dystrophy Association, Washington, DC 20006, USA; 5 Department of Pediatrics, University of California, San Francisco, CA 94143, USA; 6 Departments of Law and Medicine (Obstetrics and Gynecology), University of Michigan, Ann Arbor, MI 48109, USA; 7 Department of Bioethics, School of Medicine, Case Western Reserve University, Cleveland, OH 44106, USA; 8 Miller School of Medicine, Hussman Institute for Human Genomics, University of Miami, Miami, FL 33136, USA

**Keywords:** Duchenne, muscular dystrophy, newborn screening, carrier, X-linked disease

## Abstract

Duchenne muscular dystrophy (DMD/Duchenne) is one of the ten most severe and common pediatric genetic diseases and affects an estimated 1 in every 5000 male births. While Duchenne is a 100% fatal disease, the clinical community has demonstrated that immediate identification and early clinical interventions can add years, even decades to an individual’s life span. In anticipation of the changing therapeutic landscape for the Duchenne community, Parent Project Muscular Dystrophy established a newborn screening (NBS) initiative. This initiative included a Bioethics and Legal Issues Workgroup to consider the bioethics and legal issues of NBS for Duchenne. The workgroup’s discussion focused only on Duchenne NBS and met through conference calls over a one-year period of time seeking consensus on various identified issues. This article reports on the findings and recommendations from that workgroup.

## 1. Background

In the United States newborn screening (NBS) pilots are designed to generate analytical and clinical validation data of the selected screening methodology, establish short-term follow-up algorithms to diagnose screen positive cases, and engage the community of clinicians who will care for diagnosed newborns. Due to advancements in screening technologies, disease understanding and treatments, conditions that are candidates for inclusion in a nationwide panel of disorders recommended for screening, number in the hundreds. Some of these candidate conditions present new challenges for the existing NBS system for a number of reasons including (1) molecularly targeted therapies available for a subset of the genetic variation known to cause the disease; (2) X-linked disorders in which a minority of females present with symptoms and carrier females are identified; and (3) ongoing and planned clinical trials of novel therapies that would benefit from enrollment of newborns participating in NBS pilot. We present a case study using Duchenne muscular dystrophy (DMD/Duchenne) to address these challenges and suggest solutions. 

Duchenne muscular dystrophy (DMD) [1,2,3] is the most common form of muscular dystrophy, affecting approximately 1:5000 male births. While inherited in an X-linked pattern, about 33% of all patients are due to de novo mutations and germ line mosaicism is frequently observed. Those affected typically require a wheelchair for mobility in their second decade, are profoundly debilitated by their late teens, and generally succumb to complications of weakness in their 20’s or early 30’s. Disabling mutations of the dystrophin gene causes DMD, but hypomorphic mutations of dystrophin can cause Becker muscular dystrophy, a similar disorder that manifests over a wide range of severity. Heterozygous females are generally asymptomatic though often have mildly elevated levels of creatine kinase (CK) and can manifest variable symptom ranging from mild muscle cramping to cardiac disease; rarely heterozygous females will manifest weakness across the full spectrum experienced by affected males. In the last year, the therapeutic landscape has advanced significantly with the approval of the first two pharmaceuticals for use in Duchenne in the United States and a third product approved outside the US and currently under review by the FDA. The robust pipeline of investigational therapies advancing within clinical testing represents both genotype-specific approaches, as well as therapies with broad applicability. At this time, the standard of care in Duchenne includes steroid therapy, consideration of initiation of recently approved approaches for those with eligible genotypes, and early intervention to mitigate developmental and motor delays.

Because of therapeutic advances, NBS for Duchenne has become a priority of the Duchenne community as researchers made significant progress towards new treatments and screening technologies [1,2,3,4]. The prospect that disease-modifying treatments would prove successful, and that early treatment might then maximize that benefit, led the national patient advocacy group Parent Project Muscular Dystrophy (PPMD) in 2015 to support an intensive investigation of the required components associated with a national Duchenne Newborn Screening Program [Duchenne NBS] effort to identify clinical, NBS, family, and ethical and legal issues [1]. 

The goal of this national Duchenne NBS effort was to identify the public health infrastructure and support required to enable a NBS pilot and ultimate implementation of Duchenne NBS in the United States. PPMD first established a Duchenne NBS steering committee, comprised of representatives from federal agencies, the Duchenne patient community, the Muscular Dystrophy Association (MDA), PPMD, and experts in genetics, pediatrics and neuromuscular disorders. The initial action of the steering committee was to establish six workgroups focused on various actions essential to a successful Duchenne NBS program and included: (1) Outreach to and Education of Patient Community and Healthcare Providers; (2) Follow-up and Clinical Care Considerations for Pre-symptomatically Identified Infants with Duchenne; (3) Laboratory Test Validation and Refinement and Development of Screening Algorithm; (4) Building a Longitudinal Data Resource for Follow-up; (5) Evidence Review; and (6) Bioethics and Legal Issues. These workgroups have sought a wide range of expertise, with contributions from specialists in neuromuscular disorders, NBS, genetics and pediatrics, and included representatives from federal agencies, the American College of Genetics and Genomics’ (ACMG) Newborn Screening Translation Research Network (NBSTRN), Genetic Alliance’s Babies’ First Test, the Save Babies Foundation, and Legacy of Angels. 

The Duchenne NBS Bioethics and Legal Issues Workgroup, was tasked with examining the various ethical and legal issues raised by Duchenne NBS. The goal of the workgroup was to identify how studies for Duchenne NBS and future implementation of screening by state NBS programs could be configured to maximize benefits and minimize any harm that might be associated with a Duchenne NBS program. This paper sets out the findings and recommendations from the Duchenne NBS Bioethics and Legal Issues Workgroup, whose members are Angus Clarke, M.D. Cardiff University; Thomas Crawford, M.D. Co-Director, MDA Clinic, Johns Hopkins Medicine, Aaron Goldenberg, Ph.D., Case Western Medical School and NBSTRN; Edward Goldman, University of WI and NBSTRN; R. Rodney Howell, M.D. University of Miami Medical School and MDA; Michele A. Lloyd-Puryear, M.D., Ph.D., PPMD; Kristin Stephenson, J.D., MDA; and Tracy Trotter, M.D., FAAP.

## 2. Ethical and Legal Issues

While ethical and legal issues associated with NBS and genetic diseases have been extensively discussed within the bioethics and NBS literature [5,6], there is no established formal or systematic approach that specifically assesses the ethical or legal issues or other burdens and benefits for population-based NBS pilots. Such a formal approach could be useful to researchers and to the U.S. Advisory Committee on Heritable Disorders in Newborns and Children (ACHDNC) of the Secretary, Department of Health and Human Services, that evaluates the evidence to support addition of new disease screening programs to the Secretary’s recommended uniform screening panel (RUSP) [7,8] (see ACHDNC evidence review process [9,10]). Different approaches to the evaluation and adoption of conditions for NBS, and resulting disparity in conditions screened, have been reviewed by the European Union [11,12,13] but these reports do not systematically address specific ethical and legal issues. The ACHDNC’s evidence review process does consider harms and benefits to a limited extent, but again their process focuses generally on the technical aspects of screening, diagnosis and treatment and does not include a focused and in-depth review of the considerable ethical and legal issues that may arise when considering conditions that could be addressed during NBS pilots, such as Duchenne. Given this lack of specific guidance on the approach to ethical and legal concerns, especially for an X-linked disorder in which a minority of females are affected, the Duchenne NBS Bioethics and Legal workgroup, set forth a series of key ethical and legal questions that may be applicable for all conditions under consideration for NBS, but especially for an X-linked or emerging condition. For this paper, the Workgroup discussions limited their focus to issues raised by specific characteristics of Duchenne dystrophy, including:

- Universal screening (screening both males and females) for an X-linked disorder. In an X-linked disorder, the potential burdens and benefits are very different for males and females: DMD has variable expression in females, and carrier females have available preconception options, and
- The requirements imposed by present and proposed genotype-specific therapies in a condition where only a minority of identified patients would qualify for treatment.

Notwithstanding these Duchenne-specific issues, however, it is clear that many bioethical and legal issues applicable to DMD NBS are relevant to other NBS pilots more broadly, and thus could be useful when conducting a NBS pilot for other conditions. The workgroup therefore engaged the National Institutes of Health-funded NBSTRN’s Bioethics Workgroup in the process of discussing and analyzing the identified issues. The information presented in this paper is intended to support the development of informed policy decisions and may be of use to federal agencies, professional organizations, industry, academic institutions, health-care providers, and the general public.

## 3. Duchenne NBS Ethical and Legal Questions for Pilot Studies and Evidence Review

The Duchenne NBS Bioethics and Legal Issues Workgroup first focused upon the key question of whether or not to conduct Duchenne NBS. Because there had been significant progress towards new treatments and screening technologies [1,2,3] for Duchenne, the Workgroup deemed a NBS pilot is appropriate and justifiable. Workgroup discussion then shifted to whether a Duchenne NBS pilot should be targeted to males only or be conducted universally. With a differing perspective on the spectrum of disease in males and females (some thought the burden of disease in females was not significant enough to support NBS), the Workgroup decided to proceed with the assumption of universal NBS, acknowledging the disagreement and special concerns with universal screening for an X-linked disease. The pilot of DMD NBS could address this issue and generate new information for consideration (see later section entitled, *Question of Targeted or Universal Population Screening*). The workgroup asserted that it would be important for a pilot of DMD NBS to address this issue and generate new information for consideration before moving to whole population DMD NBS screening. 

The workgroup then framed three goals for the NBS system for Duchenne screening: (1) To enhance access to treatment trials or established therapies at an early presymptomatic time to gain maximum therapeutic benefit by leveraging use of early identification through NBS; (2) To improve the mean level of quality of care; and (3) To reduce variation in the distribution of care. In short, the DMD NBS should rest on a patient-focused and knowledge driven healthcare NBS system for individuals with Duchenne. 

Workgroup discussions also focused on the inclusion of family perspectives. The Duchenne NBS Bioethics and Legal Issues Workgroup, in line with recommendations from other review bodies, deemed it appropriate to consider the family perspectives that were included in the 2006 ACMG NBS Expert Group criteria [14]. The evidence review process used by the ACHDNC for recommending addition of conditions to the RUSP is based on the process outlined by the ACMG NBS Expert Group used to develop its recommended screening panel that was subsequently adapted by the ACHDNC as the RUSP. The criteria used in the ACMG NBS Expert Group’s evidence review process, considered and included family issues when evaluating whether a condition is appropriate for NBS. Currently, most US state NBS programs abide broadly by what is known as the Wilson–Jungner criteria [15]. These criteria were published in 1968 by the World Health Organization to address issues related to screening for adult onset common chronic conditions “that carry serious threats to the community if not detected and treated at an early stage”. At that time, the Wilson–Jungner criteria were not intended for NBS programs, and most importantly these criteria do not consider parent or patient perspectives that were included in the ACMG NBS Expert Group’s in its criteria. While general screening criteria, such as Wilson and Jungner, are useful in assessing the potential net benefits of a screening program, they do not delineate adequately the potential ethical and legal questions of screening for newborns, families, and society. The ACHDNC considered the limitations of the Wilson–Jungner criteria and added consideration of the net balance of benefit and harm and family perspectives as criteria [10]. Recent work by the European Union toward harmonization of European NBS policies also has recommended that patients’ and parents’ organizations should be involved in NBS decision-making [12].

**Figure 1:**
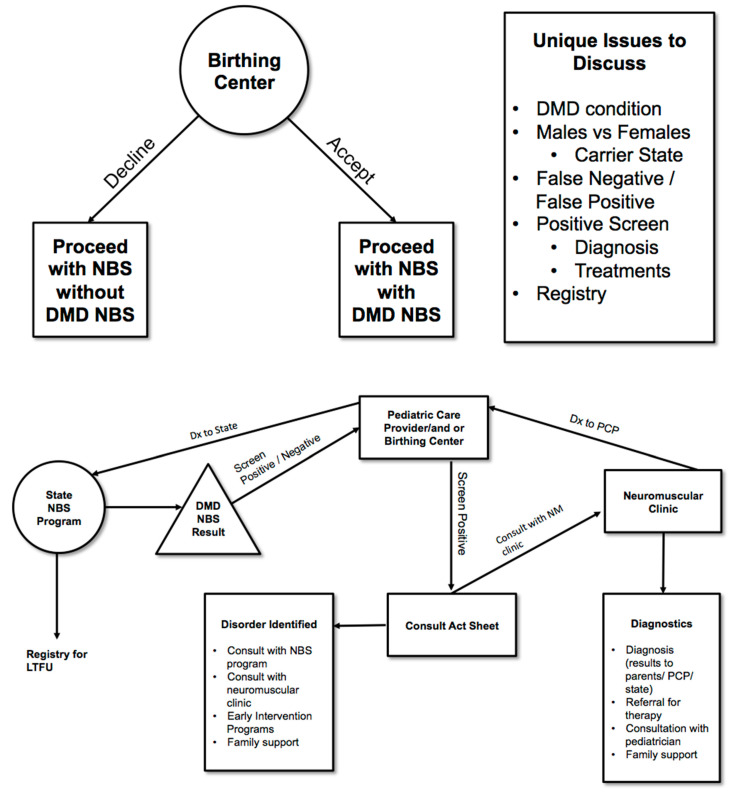
Informed Consent Process for DMD NBS.

Based on its discussions and review of the ACHDNC and ACMG approaches for evidence review and inclusion of ethical and legal issues and family perspectives, the Duchenne NBS Bioethics and Legal Issues Workgroup recommends the following ethical, legal and programmatic issues be considered in DMD NBS pilot studies and the subsequent evidence review:

1. How advocacy groups can best be engaged meaningfully, ensuring that patient perspectives are integrated into the evaluation and evidence review processes.
2. How to design the parental permission process when the range of phenotypic variation associated with positive screens is high or when benefits and harms are not completely known.
3. Consideration of issues surrounding universal versus gender-targeted screening for X-linked conditions.
4. Assessment of benefit should include impact of minimizing the diagnostic odyssey for patient and family alike, and other salutary effects to the family including that of enabling non-medical interventions.
5. Consideration of special difficulties that arise with identifying carrier status—Should Duchenne NBS include reporting of carrier status and if so then when, where and how to report? The Duchenne Workgroup recommended developing specific protocols to address these issues (See Figure 1).
6. Consideration of incidental findings—Should Duchenne NBS include reporting of findings incidental to the screen or to the genetic analysis of the second tier testing and if so, when, where and how to report these findings? Incidental findings secondary to an elevated CK may include, for example, a dystrophy other than Duchenne. This secondary condition/incidental finding would be ruled out as Duchenne with subsequent genetic analysis and appropriate referral (See Figure 1).
7. Role of industry in conducting pilots for newborn screening, whether for evaluating clinical devices for screening and diagnoses or for therapy and clinical trials—How does industry involvement in pilots affect pricing? How does industry involvement in pilots affect access to treatments post clinical trial? What are the ethical and legal issues if enrollment in a treatment trial is dependent on NBS?

## 4. Role of Advocacy Organizations in the NBS System

The Duchenne NBS Bioethics and Legal Issues Workgroup recognizes that there are ethical and legal questions regarding the appropriate role for advocacy organizations in the development of NBS policies, pilot studies and the nomination process for adding new conditions to the RUSP. As patient and parent preferences always have been a part of the development of NBS programs and policy development [10,11,12,13,14], the authors acknowledge that the lived experience, personal stories, and knowledge about development of a rare disease are valuable to understanding the impact of a condition for newborns and families, as well as the potential benefits of early detection (even in the absence of established treatment). 

In addition, within advocacy groups of the rare disease community, participation in and even driving research specific to their disease has been a high priority. Some advocacy groups that have provided major funding for clinical initiatives and research have supported methodologically sound and rigorous studies for conditions for purposes of consideration of addition to the RUSP. Patient advocacy groups have formed disease registries, have generated scientific publications, and importantly have funded research that has led to the development of treatments and understanding of disease. These efforts have been the result of collaboration among advocates, patients, researchers and clinicians. While biases can be injected in any process, collaborations with lay advocates offer unique vantage points that can enrich the process of collaboration for research and program and policy development. 

Therefore, the Duchenne NBS Bioethics and Legal Issues Workgroup recommends:

- Lay-advocates and researchers should continue to collaborate in research and policy development including the design of research studies.
- Efforts should be made to design patient-centered studies when structuring pilots or clinical trials. Pilots or clinical trial designs should not focus solely on the technical aspects of screening but the designs should include a broader range of issues to assure a patient-centered approach. Lay advocates, as with academic researchers, should disclose funding from drug companies or other entities that might contribute to the perception of bias.
- Lay-advocate leaders should guide their actions with transparency, clearly distinguishing between instances when they are speaking for themselves, for other lay-advocates or for their organization.

## 5. Considerations of Universal versus Targeted Screening and Reporting of Carriers and Incidental Findings

Duchenne results from hemizygous mutations on the X chromosome of males, and when inherited is acquired from a heterozygous mother. While both male and female newborns can inherit the dystrophin mutation, the impact is very different in the two sexes. Males having only one X chromosome lack a normal second dystrophin gene to produce the necessary protein, while females generally manifest little or no symptoms. There is a range of carrier manifestation in females, however, ranging from mild increase in measured levels of the muscle-specific plasma (creatine kinase) CK to rare females who manifest the full Duchenne phenotype. An increase in maturity-onset cardiomyopathy is sufficiently common and severe that periodic cardiac assessments of known adult carrier females are recommended [16].

The high incidence of Duchenne is in part related to the very large dystrophin gene, vulnerable to mutation as a consequence of its size, as well as the capacity of an X-linked condition to propagate in families despite reduced reproductive fitness of affected males. Disease-causing mutations in the dystrophin gene involve the full range of errors, including large and small deletion and insertions as well as sequence changes. Present screening protocols assess the levels of CK or CK specific to skeletal muscle (CK MM) in blood with follow-up including a targeted assessment of the dystrophin gene via DNA-based molecular methods. The nature of an identified dystrophin mutation often affords some clinical insight into prognosis—Duchenne results from complete absence of the dystrophin gene, where the milder “Becker” muscular dystrophy (BMD) results from various partial impairments of dystrophin abundance or function. One approach to therapy involves manipulation of specific Dystrophin mutations that enable restoration of partial gene function, thus hopefully converting a boy destined to manifest the severe DMD phenotype to that of the milder BMD disorder. Because the use of these therapies is-based upon the exact disease-causing mutation that is present, molecular genetic testing of the *DMD* gene of those infants with elevated CK values in the newborn screen will be necessary for access to appropriate molecular therapies when possible. 

Identification of the Duchenne causing mutation in a newborn prompts genetic testing of the mother and, potentially, cascade testing of the other family members. In approximately 2/3 of identified individuals found to have Duchenne, the dystrophin mutation is present in the mother. However, a mother may screen negative as 1/3 of the Duchenne cases are because of de novo mutations. One mechanism of de novo mutations identified is germ-line mosaicism. This mechanism has important implications for the counseling of DMD families.

The Duchenne NBS Bioethics and Legal Issues Workgroup therefore focused its recommendations for a pilot on the following two areas of concern: (1) targeted versus universal screening, and (2) designing follow-up infrastructure that facilitates proper diagnosis and treatment, including genetic testing and adequate counseling education for parents and health care providers.

### 5.1. Question of Targeted or Universal Population Screening

In the United States, NBS screening programs are administered by the states and all states conduct their programs in a gender neutral or universal fashion, i.e., all babies are screened for the State’s recommended NBS panel [17,18]. Designing a Duchenne NBS program that screens males only (as some other countries have done), would be a departure for NBS programs in the United States, and carries the risk of missing boys (due to errant labeling of sex on the NBS card) and might be considered cumbersome and expensive for the State NBS program. Some workgroup members thought that the tenet of social equity underlying a universal approach might bring unintended consequences, such as likely relatively common misidentification of a screen negative female as not being a carrier because of poor resolution of carriers from normal on CK values alone. 

Upon weighing these contrasting points of view, the Duchenne Bioethics and Legal Issues Workgroup recommends:

- The Duchenne NBS pilot should be designed to conduct universal screening of newborns while concurrently exploring the many ethical and legal issues raised by screening for an X-linked disease. The issues include those raised in the above section as well as the following section addressing follow-up practices. An arm of the pilot might consider evaluation of parental attitudes on gender-targeted versus universal screening.

### 5.2. Follow-Up of Infrastructure for Families and Screened Positive Infants and How to Report Carrier Status and Incidental Findings

Some of the most difficult issues in NBS for Duchenne will be the follow-up care, for not only the boys screening positive, but also for girls, mothers and the families. The Workgroup proposes the infrastructure and flow of information illustrated in Figure 1 could be used to address many of the issues raised by Duchenne NBS. The Workgroup acknowledges also that the ideal pilot of DMD would have sufficient duration to fully assess health outcomes including collecting information related to diagnosis after a positive screen, referral to the appropriate clinician for care, timing and type of treatment. In 2010, international clinical care standards for Duchenne and Becker (DBMD) were published [19] following an international expert consensus process convened by the U.S. Centers for Disease Control and Prevention (CDC). These care standards have been translated into more than 40 languages, converted into a “family-friendly version” and widely disseminated to clinical care centers throughout the world. In 2017, the CDC DBMD Care Considerations were updated [20,21,22] and implementation efforts are underway. The Duchenne space is fortunate that there exist international, coordinated Duchenne care networks supported by a multitude of advocacy organizations all anchored to published care standards. While this network exists, there will still be challenges surrounding the referral of those patients to appropriate care centers and ensuring that published care standards are updated in a timely way to reflect the advent of NBS in the Duchenne community.

Since all of the current disease modifying treatments for Duchenne depend on knowing the specific genetic defect, it is essential, and ethically required, that all screen positive infants have access to genetic testing (to define their specific mutations) and access to appropriate health care disease expertise, in order that an appropriate treatment can be provided. In addition, since the condition is X-linked, important family testing issues exist in regard to carrier status: testing mothers; testing female siblings of affected infants; and testing extended family.

In establishing a Duchenne NBS pilot, the ethical and legal issues surrounding genetic testing of various family members should be evaluated. Recommendations by professional bodies are generally consistent: the majority of guidelines recommend against carrier testing of minors unless there is medical benefit to the child [23,24,25,26]. Analysis of the dystrophin gene will be a necessary second tier step in a DMD NBS program. Some screen positive infants will have other forms of muscular dystrophy, and some will have DMD/BMD caused by mutations not identified in the genetic test. For mothers of screen positive infants with an identified dystrophin mutation genetic testing is relatively simple. Some women who are carriers will test as normal on this test, because of germ-line mosaicism, wherein the mutation is present in the ovary tissue but not in blood. In these rare circumstances the sisters of affected males also may be carriers even if their mother is shown not to be a carrier. Various mutation etiologies must be addressed during genetic testing as part of counseling and education of individuals affected by Duchenne and their parents.

Finally, identifying threshold CK (or CK MM) value to identify affected males efficiently is very different than that used to identify carrier females. A normal CK, or below threshold value, does not provide assurance of non-carrier status in a NBS program. The potential exists for misunderstanding of the power of a negative screen.

Therefore, the Duchenne Bioethics and Legal Issues Workgroup recommends the pilot must:

- Evaluate whether all infants identified with Duchenne or as carriers have received adequate follow-up including diagnosis after a positive screen, referral to appropriate clinical care centers, delivery of best practice treatment and management across the lifespan.
- Ensure that the partnering state NBS program have a short-term follow-up plan in place to ensure screen positive newborns receive proper referral to appropriate center for diagnosis and referral for treatments. This short-term follow-up program should:
  ▯ Have diagnostic capacity in place that includes genetic sequencing, or referral for genetic sequencing, for screen positive infants.
  ▯ Consider cascade genetic testing of both families and extended families. The project should have funds necessary to carry out appropriate genetic testing for diagnosis and in the family.
  ▯ Include appropriate triage for infants with incidental findings such as sex chromosome disorders or other neuromuscular diseases.
  ▯ Protocol for reporting of findings incidental to an elevated CK. The project should determine the appropriate CK (immunoassay) cut off to minimize false negatives and detection of carriers. A CK level needs to be determined that will identify all infants with Duchenne, and at the same time determine how frequently, if at all, female carriers are identified.
  ▯ Determine an appropriate educational process that addresses public health and health care provider and parental genetic literacy about genotype/phenotype relationship/X-linked disorders/sex chromosome disorders.

## 6. Conclusions

NBS around the world is rapidly expanding to include new conditions as technical, clinical and treatment knowledge advances. Historically pilots establish the evidence base and understanding needed for ultimate population-based screening, but they now need to evolve to also address ethical and legal issues across the lifespan. Using DMD as a case study, we presented a systematic approach to the evaluation and consideration of ethical and legal issues in the context of conducting a NBS pilot. We worked to address relevant these issues for Duchenne NBS, but this is not intended to be inclusive of all potential issues that may arise within a Duchenne NBS pilot. As we move forward toward implementation of Duchenne NBS, however we will need to address these issues, and design the public health and health care service infrastructure needed to ensure that we do not lessen the benefit of the early identification of newborns with Duchenne.
